# Monitoring of somatic parameters at outpatient departments for mood and anxiety disorders

**DOI:** 10.1371/journal.pone.0200520

**Published:** 2018-08-21

**Authors:** Mirjam Simoons, Hans Mulder, Bennard Doornbos, Robert A. Schoevers, Eric N. van Roon, Henricus G. Ruhé

**Affiliations:** 1 Department of Clinical Pharmacy, Wilhelmina Hospital Assen, Assen, The Netherlands; 2 Department of Psychiatry, Interdisciplinary Centre for Psychopathology and Emotion regulation, University of Groningen, University Medical Centre Groningen, Groningen, The Netherlands; 3 Department of Pharmacotherapy, -Epidemiology & -Economics, Department of Pharmacy, University of Groningen, Groningen, The Netherlands; 4 Mental Health Services Drenthe, Assen, The Netherlands; 5 Department of Clinical Pharmacy and Clinical Pharmacology, Medical Centre Leeuwarden, Leeuwarden, The Netherlands; 6 Department of Psychiatry, Warneford Hospital, University of Oxford, Oxford, United Kingdom; Department of Psychiatry and Neuropsychology, Maastricht University Medical Center, NETHERLANDS

## Abstract

**Introduction:**

Somatic complications account for the majority of the 13–30 years shortened life expectancy in psychiatric patients compared to the general population. The study aim was to assess to which extent patients visiting outpatient departments for mood and anxiety disorders were monitored for relevant somatic comorbidities and (adverse) effects of psychotropic drugs–more specifically a) metabolic parameters, b) lithium safety and c) ECGs—during their treatment.

**Methods:**

We performed a retrospective clinical records review and cross-sectional analysis to assess the extent of somatic monitoring at four outpatient departments for mood and anxiety disorders in The Netherlands. We consecutively recruited adult patients visiting a participating outpatient department between March and November 2014. The primary outcome was percentage of patients without monitoring measurements. Secondary outcomes were number of measurements per parameter per patient per year and time from start of treatment to first measurement.

**Results:**

We included 324 outpatients, of whom 60.2% were female. Most patients were treated for depressive disorders (39.8%), anxiety disorders (16.7%) or bipolar or related disorders (11.7%) and 198 patients (61.1%) used at least one psychotropic drug. For 186 patients (57.4%), no monitoring records were recorded (median treatment period 7.3 months, range 0–55.6). The median number of measurements per parameter per year since the start of outpatient treatment for patients with monitoring measurements was 0.31 (range 0.0–12.9). The median time to first monitoring measurement per parameter for patients with monitoring measurements was 3.8 months (range 0.0–50.7).

**Discussion:**

Somatic monitoring in outpatients with mood and anxiety disorders is not routine clinical practice. Monitoring practices need to be improved to prevent psychiatric outpatients from undetected somatic complications.

## Introduction

Patients with a severe mental illness (SMI), including patients with bipolar disorders and major depressive disorder, have a 13–30 year shorter life expectancy compared to the general population [1]. The majority (about 60%) of this excess mortality can be explained by somatic co-morbidity like respiratory, cardiovascular, nutritional and/or metabolic diseases [1–4]. Several factors may contribute to this increased risk of somatic morbidity and mortality, such as an unhealthy lifestyle and disparities in health care access that are associated with mental illness [1,5]. In addition, the use of psychotropic drugs may cause and/or increase the vulnerability of psychiatric patients to somatic complications due to adverse effects [1,6].

In order to detect somatic complications and psychotropic drug-induced adverse effects, several guidelines and consensus documents have suggested to monitor essential somatic parameters as part of routine clinical practice in among others patients with schizophrenia, bipolar disorder and major depressive disorder [7–10]. Similarly, guidelines have been published for somatic monitoring during the use of specific classes of psychotropic drugs [7–11]. Next to the recognition that serum lithium levels, renal function and thyroid function should be monitored during lithium therapy, more recently metabolic monitoring during antipsychotic therapy has been advocated [7,9,11]. For patients treated for major depressive disorder only recently the first consensus document on somatic monitoring has been published in which a number of baseline and antidepressant-specific follow-up tests are recommended, in part depending on patient vulnerability characteristics [8,12]. Indeed, for antidepressants debate exists, e.g. regarding the necessity and appropriate frequency of monitoring of the electrocardiogram (ECG), notwithstanding a recent FDA warning [13,14].

In contrast to the expected clinical relevance of somatic monitoring of patients with SMI and the availability of the above-mentioned guidelines, several studies have shown poor adherence to these guidelines. For example, monitoring of serum lithium level, renal function and thyroid function in patients with bipolar disorder using lithium in the United Kingdom showed only 30–55% compliance to the available guideline [15]. A meta-analysis of 39 studies on metabolic screening in patients with predominantly schizophrenia or related disorders using antipsychotics, showed that routine baseline metabolic screening before start of pharmacotherapy was suboptimal [16]. Given the high prevalence of somatic co-morbidities (e.g. metabolic syndrome, pooled prevalence of 32.6% in a large cohort of SMI patients (N = 52,678) [17]), suboptimal monitoring might put patients at considerable risk for iatrogenic harm (i.e. harm resulting from treatment by a health care professional), regardless of the specific psychiatric diagnosis.

Metabolic disturbances and other somatic complications are not limited to patients with schizophrenia or patients using antipsychotics. Mood disorders are increasingly treated with combinations of lithium, antipsychotics, mood stabilizers and antidepressants. Therefore, these patients are at risk for somatic complications as well. In this study, we investigated to which extent patients visiting outpatient departments for mood and anxiety disorders were monitored for relevant somatic comorbidities and (adverse) effects of psychotropic drugs–more specifically a) metabolic parameters, b) lithium safety and c) ECGs—during their treatment.

## Materials and methods

### Design and setting

We performed a retrospective clinical records review and cross-sectional analysis to assess to which extent somatic monitoring had been performed in outpatients with mood and anxiety disorders. The study was conducted at four different outpatient departments for mood and anxiety disorders in the Northern part of the Netherlands. Three departments were part of two large secondary mental health care institutions and one department was part of an academic hospital. One of the three community departments partly used paper medical records for registration of somatic monitoring measurements, the others used electronic medical records.

### Study population

We included patients if they were 18 years or older and had visited the participating outpatient department for the first time after January 1^st^, 2010 (source population). We applied the latter inclusion criterion to ascertain that the observation period for every participant started after the introduction of several guidelines for somatic monitoring of psychiatric patients (among others antipsychotic-induced metabolic disturbances and lithium-induced side effects) [9–11,18].

We recruited patients consecutively when they visited the participating outpatient departments between March and November 2014. We provided complete verbal and written information about the study, and obtained written informed consent for the use of their medication data from the community pharmacy and the use of their medical (psychiatric) history, laboratory results and results of other measurements from the mental health care institution for the research purposes of this study. When we would doubt whether the patient understood the information or we would consider the patient impaired to consent (e.g. low IQ), we a priori would have abstained from including the patient. However for none of the patients there was any indication of impairment to consent. The independent medical ethics committee in Leeuwarden, the Netherlands (rTPO Leeuwarden; RTPO 918c), waived formal review and approval of the study protocol since participants were not subject to procedures, nor were they required to follow rules of behaviour for this study.

### Outcome measures

We defined the observation period as the period between the start of treatment by the primary care giver (recorded in the medical record) and the inclusion (index) date.

Our primary outcome was the percentage of patients without relevant somatic monitoring measurements in their medical records during their outpatient treatment.

Our secondary outcomes were the number of measurements per somatic monitoring parameter per patient per year, as recorded in the medical records during the observation period for: I. all monitoring parameters together and, separately, II. metabolic monitoring parameters and the metabolic syndrome in the total study population, III. metabolic monitoring parameters and the metabolic syndrome in participants that used atypical antipsychotics, IV. lithium serum concentration, thyroid stimulating hormone (TSH) and kidney function in patients that used lithium, and V. ECG in the total study population. Another secondary outcome was the time from the start of treatment to the first monitoring measurement during the observation period.

### Assessment of study parameters

We defined an somatic monitoring parameter as any parameter that can be measured as part of a physical examination (e.g. blood pressure, weight or ECG) or laboratory measurements (e.g. glucose- or creatinine concentration in blood or urine or a full blood examination). We checked for records of monitoring measurements in the relevant sections of the patient’s medical records. All records on somatic monitoring measurements that were entered in the medical records were considered; this could be data generated by health care providers from the mental health care institution itself or communicated by other health care providers involved with the patient, as long as these measurements were retrievable from the medical records–either automatically from for example a laboratory system, or manually entered in the designated fields. According to the National Cholesterol Education Program Adult Treatment Panel III criteria, we considered the following criteria for assessment of the metabolic syndrome: waist circumference, triglycerides, high density lipoprotein (HDL), blood pressure, and glucose [19].

Medication records for each patient were retrieved from the community pharmacy for the observation period. We defined the use of a psychotropic drug as ‘a patient’s prescriptions for that psychotropic drug covering at least 80% of the observation period according to the community pharmacy records’. We specifically looked at the use of antidepressants, antipsychotics and mood stabilizers (i.e. lithium, sodium valproate, lamotrigine, carbamazepine, topiramate, and levetiracetam). These drug classes were chosen because of the availability of guidelines or consensus documents for somatic monitoring of patients treated with these drugs or for the associated psychiatric diseases (e.g. antipsychotics [11], major depressive disorder [8], bipolar disorder [9], schizophrenia [10]).

### Statistical analysis

We performed descriptive and statistical analyses using Excel 2013 (Microsoft, Redmond, Washington, USA) and IBM SPSS (version 20 for Windows; IBM Corp., Armonk, New York, USA). We report medians when distributions are non-normally distributed. For comparison of differences in dichotomous and categorical variables, we used Chi square tests and for comparison of differences in continuous variables we used t-tests. Differences were considered statistically significant when p<0.05.

For comparison of categorical and continuous variables between academic and community settings or between patients with and without monitoring measurements, we used χ^2^ tests or Fisher’s exact tests as appropriate and t-tests, respectively. We report medians (range) when distributions are not distributed normally. We investigated the registration of monitoring parameters (yes/no) and the number of monitoring measurements during the observation period for differences per setting (both the four outpatient departments separately and the academic/community departments) in univariate logistic regression models and univariate linear regression models. In all analyses, we used a p<0.05 significance level.

## Results

### Participants

**Fig 1:**
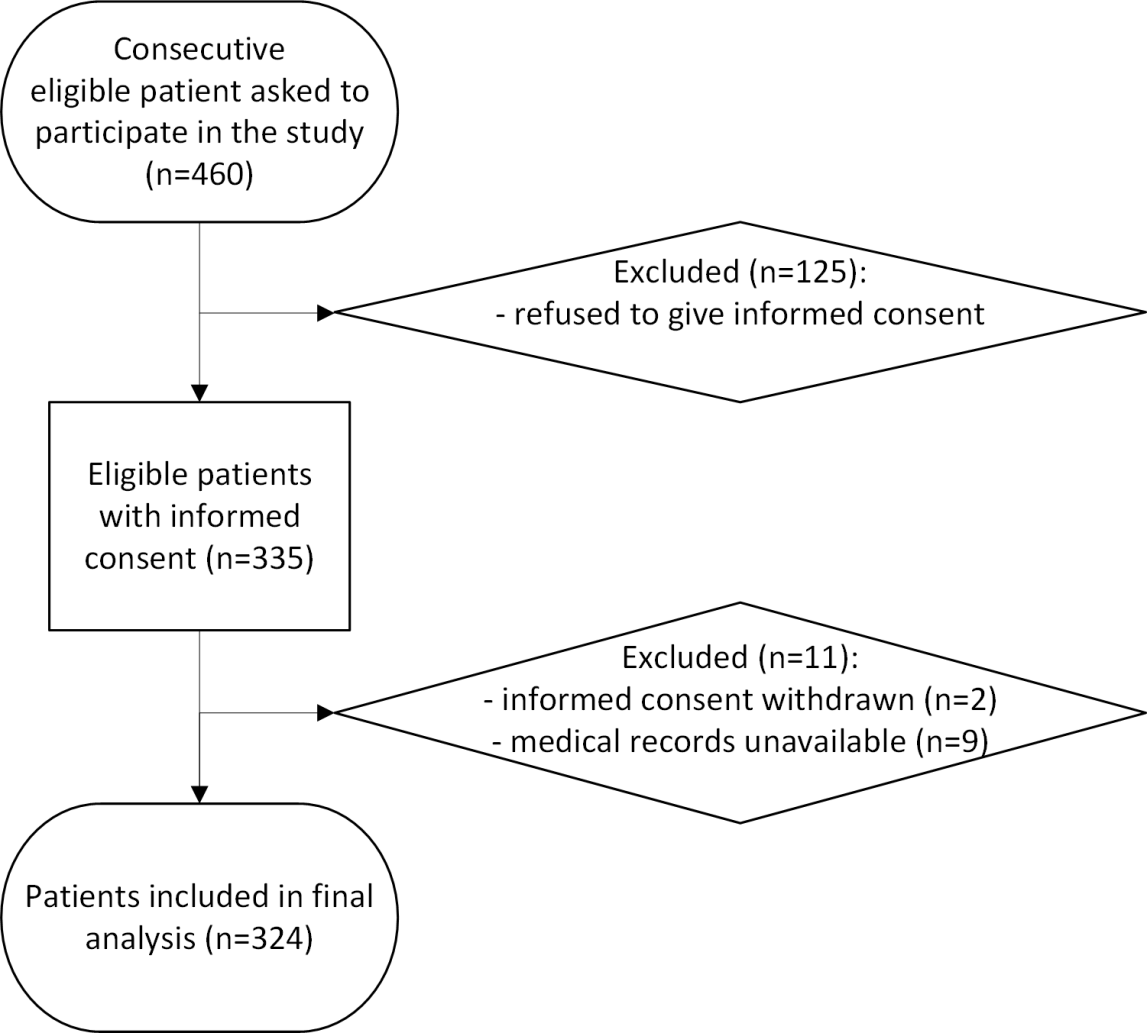
Flow diagram of patient inclusion.

During the study period between March 2014 and November 2014, we consecutively asked 460 eligible patients to participate in the study at each of the four locations. Of these, 335 patients gave written informed consent. In Fig 1 the flow diagram of patient inclusion is shown. The consent rate did not differ statistically between the four centres (p = 0.543). Eleven patients had to be excluded for subsequent analyses: two because they withdrew their informed consent in second instance and nine because their medical records were unavailable.

Table 1 summarizes the characteristics of the total study population (n = 324) and of the patients treated in the academic and community settings, as obtained from the clinical records from the mental health care outpatient department and the medication records from the community pharmacy. Of all participants, 60.2% were female, as can be expected in mood and anxiety disorders. Most patients had low or medium educational levels (76.6%) and had been treated for depressive disorders (39.8%), anxiety disorders (16.7%) or bipolar or related disorders (11.7%). During their observation period, 198 subjects (61.1%) used at least one psychotropic drug during at least 80% of the observation period.

**Table 1:** Characteristics of the total study population and the populations from academic and community settings.

Characteristic	Value^a^^,^^b^		
	*Total (n = 324)*	*Academic setting (n = 54)*	*Community settings (n = 270)*
Sex			
Female	195 (60.2%)	34 (63.0%)	161 (59.6%)
Age (years)	43.6±12.4	40.8±13.8	44.2±12.0
Educational level			**
Low	88 (27.2%)	7 (13.0%)	81 (30.0%)
Medium	160 (49.4%)	24 (44.4%)	136 (50.4%)
High	74 (22.8%)	22 (40.7%)	52 (19.3%)
Unknown	2 (0.6%)	1 (1.9%)	1 (0.4%)
Duration of outpatient treatment (median (range); months)	7.3 (0.0–55.6)	16.3 (2.0–55.0)	6.3 (0.0–55.6) ***
Primary psychiatric diagnosis (DSM V diagnostic criteria)^c^			
Bipolar or related disorder	38 (11.7%)	9 (16.7%)	29 (10.7%)
Depressive disorder	129 (39.8%)	16 (29.6%)	113 (41.9%)
Anxiety disorder	54 (16.7%)	10 (18.5%)	44 (16.3%)
Other psychiatric disorder	71 (21.9%)	14 (25.9%)	57 (21.1%)
Not yet diagnosed	30 (9.3%)	5 (9.3%)	25 (9.3%)
Unknown	2 (0.6%)	0 (0.0%)	2 (0.7%)
Patients using a psychotropic drug at any stage during observation period	198 (61.1%)	26 (48.1%)	172 (63.7%) *
Of whom used: antidepressants	156 (78.4%)	20 (76.9%)	136 (78.6%)
classic antipsychotics	6 (3.0%)	0 (0.0%)	6 (3.5%)
atypical antipsychotics	42 (21.1%)	8 (30.8%)	34 (19.7%)
lithium	42 (21.1%)	7 (26.9%)	35 (20.2%)
non-lithium mood stabilizers	14 (7.1%)	1 (3.8%)	13 (7.6%)
combinations of ≥2 psychotropic drugs	49 (24.6%)	7 (26.9%)	42 (24.3%)

^a^ Data are given as number (percentage) or mean ± standard deviation unless stated otherwise.

^b^ *p<0.05, **p≤0.01, ***p≤0.001 for academic vs. community settings.

^c^ DSM Diagnostic and Statistical Manual of Mental Disorders.

### Primary outcome: Registration of monitoring information

For 186 of 324 patients (57.4%), we identified no monitoring measurements in their medical records during the median observation period of 7.3 months (range 0.0–55.6). In our logistic regression models, the lack of registration of monitoring measurements at the three community outpatient departments (62.5%, 67.8% and 58.7%) did not differ statistically between departments (p = 0.446), but was statistically different from the academic outpatient department (29.6%) both for each community department separately and grouped together (all p<0.001). These differences remained significant when we corrected for the longer observation period in the academic setting versus the community settings in multivariate logistic regression models (all p≤0.038; see Table 1).

Patients for whom monitoring measurements were reported (n = 138; 42.6%), had been in treatment for a significantly longer period on the index date compared to patients without monitoring measurement: 13.9 (range 0.66–55.0) vs. 4.9 (range 0.0–55.6) months (p<0.001). There was no difference in registration of monitoring measurements between patients with and without psychotropic drugs (46.5% vs. 36.5%; p = 0.077). However, for 106 of 198 (53.5%) patients who had used at least one psychotropic drug during the observation period, no monitoring data were recorded. For patients using antidepressants, 62.8% were without monitoring measurements, which was 16.7% for patients using classic antipsychotics, 38.1% for atypical antipsychotics, 9.5% for lithium and 21.4% for other mood-stabilizers.

### Secondary outcomes: Monitoring frequency and timing of monitoring

The median number of monitoring measurements per monitoring parameter per patient with monitoring measurements per year was 0.31 (range 0.0–12.9). Our linear regression analyses showed that this number was not different between the four outpatient departments nor between the academic and community settings (all p>0.254).

**Fig 2:**
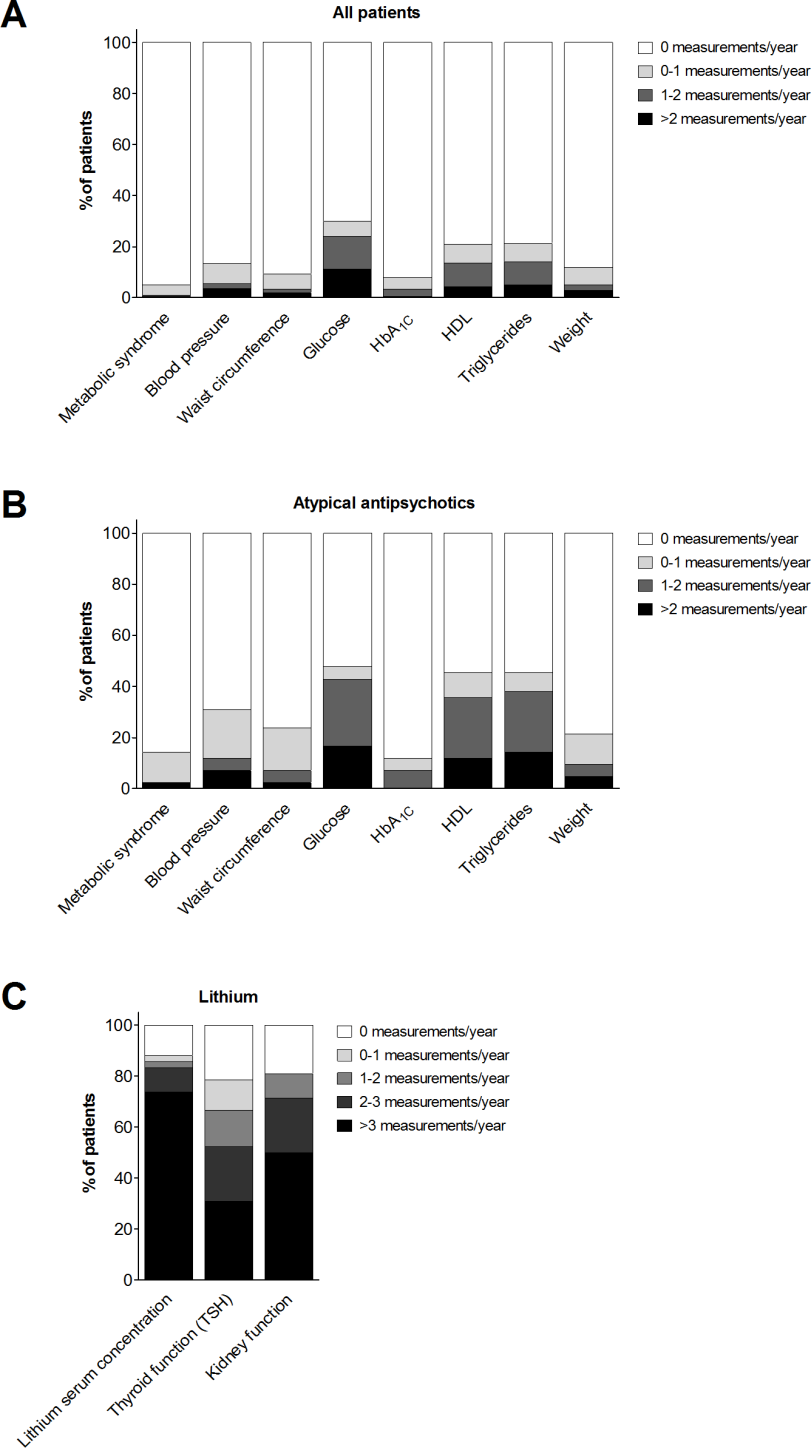
Metabolic monitoring and lithium monitoring in total and subsets of study population. Monitoring frequencies of metabolic parameters in (A) the total study population (n = 324, of whom 61.1% used psychotropic drugs) and (B) in patients using atypical antipsychotics (n = 42) and (C) lithium serum concentration, thyroid function and kidney function in patients using lithium (n = 42). Diagnostic criteria for the metabolic syndrome were: registration of measures of waist circumference, triglycerides, High Density Lipoprotein (HDL), blood pressure, and glucose, according to the National Cholesterol Education Program Adult Treatment Panel III criteria [19].

Fig 2 shows the frequency of monitoring for metabolic parameters and the metabolic syndrome in the total study population (panel A) and in participants that used atypical antipsychotics (n = 42; panel B). The percentage of patients without metabolic measurements was 66.4% for all patients and 38.1% for patients using atypical antipsychotics. Patients who used atypical antipsychotic drugs, were monitored more often than the total population for all metabolic parameters. In addition, Fig 2C displays the frequency of monitoring of the lithium serum concentration, TSH and kidney function in patients who used lithium (n = 42). Less than two measurements of lithium serum concentration, thyroid and kidney function per year (minimum in the Dutch guideline for Bipolar Disorders) were performed in 16.7%, 47.6% and 28.6% of patients using lithium, respectively (median treatment duration 15.1 months (range 1.0–49.1). Measurement of at least one ECG was performed in ten patients (3.1%; range 1–6 measurements), with an average observation period of 26.1 months (standard deviation 12.7). Finally, the median time to the first monitoring measurement per parameter for patients with monitoring measurements since the start of treatment was 3.8 months (range 0.0–50.7).

## Discussion

This naturalistic, non-intervention study shows that monitoring of somatic parameters at outpatient departments for mood and anxiety disorders is not implemented in daily clinical practice. Compared to the total study sample, a slightly higher number of metabolic measurements was found in the subsample using atypical antipsychotics. Lithium monitoring rates in the subpopulation using lithium were considerably better. However, for 57.4% of all patients, there were no records of monitoring measurements. Moreover, the median time to the first measurement was almost four months after the initiation of outpatient treatment, indicating that a somatic screening at the first appointment is not part of routine clinical practice. Improvement of these monitoring practices provides an opportunity to ensure that somatic complications and adverse drug effects are detected and treated in order to reduce the risk of iatrogenic harm.

### Somatic monitoring in psychiatry

Our results are in agreement with several studies on monitoring in psychiatric outpatients. For example, in a large benchmarking audit from The United Kingdom in lithium-treated patients, no weight had been recorded in 72% of patients, no tests had been performed on kidney and thyroid function in 19%, and 18%, respectively, and no lithium serum concentration had been taken in 9% [15]. A recent study in underserved adults with SMI taking antipsychotic drugs showed that in only 30.1% of patients adequate diabetes-specific screening (glucose serum test or HbA_1C_ test) was performed [20]. With 90–100% screening for hypertension, diabetes and dyslipidaemia in HIV-patients compared to 40–70% in psychiatric outpatients taking antipsychotics, somatic monitoring frequencies were found to be considerably higher in other fields of outpatient medicine, suggesting a particular problem of poor monitoring in psychiatry [21]. Considering the similarity between these reports and our study, we believe our results are generalizable to other parts of The Netherlands and Europe, although replication is warranted.

Somatic monitoring is important in patients with mood and anxiety disorders because these patients are at risk for cardiovascular, nutritional and metabolic comorbidities, irrespective of the use of psychotropic drugs [1,8,22]. Somatic complications account for the majority of the excess mortality which translates to a 13–30 year shorter life expectancy in the SMI population compared to the general population [1]. The use of psychotropic drugs like antipsychotics or mood stabilizers but also specific antidepressants further increases the risk of somatic complications as a result of, among others, drug-induced disturbances in metabolic parameters, liver function and ECG [6,8,23,24]. Although the effectiveness of somatic monitoring on treatment outcomes has not been well established [25], it seems logical to monitor these known somatic complications of mood and anxiety disorders and adverse effects of psychotropic drugs, especially in patients with a high prevalence or a high risk of morbidity or mortality [26,27]. Importantly, this low rate of monitoring is in contrast with the SMI patients’ perceived need to have their somatic health screened and monitored, as their ability to survey their own physical health interest is reduced [28].

### Lithium, metabolic and ECG monitoring

Lithium monitoring was considerably better than metabolic monitoring, although for both sets of parameters improvement could still be achieved. For patients using antipsychotics or lithium, national and international consensus documents and guidelines on monitoring are available [7,11]. A possible explanation is that psychiatrists in the field of mood and anxiety disorders might not be aware of monitoring guidelines for antipsychotic use, especially when dosages of antipsychotics are low and duration of use is relatively short, or that the necessity of lithium monitoring has been more longstanding acknowledged in consensus documents or guidelines compared to metabolic monitoring during antipsychotic use. Interestingly, ECG measurements were performed in only 10 out of 324 patients. However, we cannot value this finding in terms of guideline concordance or clinical necessity, as there is no clear or uniform set of criteria for measuring the ECG and we could logistically not verify many of the various risk factors in our patient population.

### Baseline somatic screening

An interesting finding of this study is that the first monitoring measurement was performed approximately four months after the start of treatment. This median four month delay suggests that a baseline somatic screening is not part of daily routine. Such a baseline measurement before starting any treatment is important to (1) clarify the (type of) psychiatric disorder and assist in (drug) treatment selection (2) assess the effect of co-morbidities on the psychiatric diagnosis (3) determine risk factors that may increase susceptibility to adverse effects, and (4) establish intra-individual references for any (aberrant) test result of monitoring thereafter [8]. Without a baseline measurement, the treating psychiatrist cannot determine whether aberrant measurements during treatment are the result of (drug) treatment. Reasons for not performing a baseline screening may include limited access to laboratory facilities for physicians and/or patients, insufficiently experienced or educated mental health care professionals or seemingly good somatic health (in young patients). These factors may also play a role in the lack of somatic monitoring in general.

An important issue that has to be investigated further is which patients have to be measured for which parameter and how frequently, which might depend on an a-priori risk-profile instead of a one-size fits all approach. A minimum set of monitoring recommendations should be established for groups of patients depending on relevant risk factors that is cost-effective and is readily applicable in routine clinical practice. It is essential to find the right balance between costs and yield of relevant aberrances, thereby creating a set of parameters that gives monitoring information to support the clinician in making treatment decisions without producing many test results that are not hypothesis-driven or based on a specific increased risk.

### Implementation of somatic monitoring

In previous research, the introduction of new guidelines, consensus statements, or (national) quality improvement programs alone appeared to be minimally effective in improving monitoring rates [16,29–31]. Therefore, to our opinion, the implementation of a more structured monitoring program is warranted in which somatic monitoring is ensured as part of routine clinical care [32]—ideally in close collaboration between physical and mental health professionals. The differences found between academic and community mental health providers suggest that collaboration between centres may also be beneficial to enhance broader implementation. A computer reminder system has been shown to be effective in supporting laboratory monitoring of psychiatric outpatients using antipsychotics [33]. In the northern part of The Netherlands, we developed the innovative care path ‘Monitoring Outcomes of Psychiatric Pharmacotherapy (MOPHAR)’, which is currently actively implemented as a restructured routine practice for outpatient care teams. In this program, somatic monitoring of psychiatric outpatients is incorporated in routine clinical practice at the outpatient department. Primary objective of this program is to prevent, monitor and treat somatic co-morbidities and adverse effects of psychotropic drugs. A nurse conducts and coordinates general somatic screenings with each patient at the first appointment and yearly thereafter. In addition, recommended monitoring (among others of therapeutic effect and somatic adverse effects of psychotropic drugs) is performed according to pre-specified protocols per drug used as determined by regular medication reconciliation [34]. Mental health care providers have immediate access to this up-to-date information and minimal burden to pursue these protocols. This program will be further investigated for effectiveness.

### Strengths and limitations

Strengths of this study are the large population and the conduct of assessments at four locations. However, a few limitations need to be considered when interpreting our results. Firstly, as we performed a retrospective review of the medical records, our results depend on their accuracy and completeness. We did not obtain additional information from health care providers or patients. Therefore, we cannot exclude that monitoring of somatic parameters was done by others, for example a general practitioner, when such external results were not accessible through the patient’s medical records at the outpatient department. Nevertheless, we intentionally designed the study to include only monitoring measurements recorded in the medical records at the outpatient departments, as this information is used for treatment decisions by the mental health care providers. Secondly, we did not systematically ascertain whether monitoring measurements were recorded before the start of treatment at the outpatient department. To address this limitation, we performed an exploratory search for laboratory data including general practitioners’ data in 50 patients in the three months before the start of their treatment. We specifically looked at blood parameters (hemoglobin, hematocrit and full blood count), electrolytes (sodium, potassium and calcium), kidney function, blood lipids (cholesterol, triglycerides, HDL and low density lipoprotein (LDL)), glucose of hemoglobin A_1C_ (HbA_1C_) and thyroid function. For one patient (2.0%), all these parameters were recorded, while for 31 patients (62.0%) none of these parameters were measured. This indicates that a baseline screening prior to start of outpatient treatment, for example performed by a general practitioner, is not routinely performed in clinical practice either. Thirdly, as mentioned before, antipsychotics in patients with mood and anxiety disorders may be used for a short period at a low dose (e.g. quetiapine 50 mg q.d.). In these patients, monitoring may have been considered irrelevant. However, only 7 out of 42 patients (16.7%) using atypical antipsychotics used low dosages (≤100 mg/day) quetiapine for less than one month (next to use of another psychotropic drug for more than 80% of the observation period in 5 patients), indicating that this alone cannot explain the relatively low monitoring frequencies in these patients. Furthermore, there is evidence that metabolic adverse effects of antipsychotics also occur during short-term use and at low dosages, thus requiring monitoring anyhow [35,36]. Finally, as we could logistically not verify somatic diseases in a valid way in these patients, we are unable to explore any associations of monitoring frequency with somatic comorbidities. We did not explore any associations with the psychiatric illnesses either (Table 1). However, our population had been referred to the outpatient clinic for worse than minor psychological complaints, warranting somatic monitoring at least once a year regardless of the specific psychiatric diagnosis or additional somatic comorbidities.

### Conclusions and suggestions for future research

In conclusion, this study shows that monitoring of somatic parameters before and during treatment in psychiatric outpatients with mood and anxiety disorders is not performed routinely in clinical practice. Although risks vary between different drugs and patients, the low frequencies of systematic monitoring might increase the risk of undetected somatic complications of the psychiatric illness or due to adverse effects of psychotropic drugs in these patients. The results of this study will hopefully increase the awareness of mental health care providers that better monitoring of psychiatric treatments poses an opportunity for improving the care for their vulnerable patients. Future studies should address whether special monitoring programs, with routine medication reconciliation and monitoring of treatment effects, somatic complications and adverse drug effects can improve monitoring frequencies at outpatient departments for psychiatric patients. These studies should also address the questions whether such monitoring programs indeed reduce the risk of prospective somatic complications in psychiatric patients and how these programs optimize the benefits of improved quality of life at the lowest cost of (too many) examinations and check-ups.
